# The Value of the Systemic Immune-Inflammation Index in Predicting Survival Outcomes in Patients with Brain Metastases of Non-Small-Cell Lung Cancer Treated with Stereotactic Radiotherapy

**DOI:** 10.1155/2021/2910892

**Published:** 2021-10-29

**Authors:** Yanan Zhang, Zeyang Chen, Feng Jin, Dong Guo, Qingqing Chen, Zhengcao Liu, Shengjun Ji, Guanqi Gao

**Affiliations:** ^1^Clinical Medical College, Weifang Medical University, Weifang, China; ^2^Linyi People's Hospital, Linyi, China; ^3^Clinical Medical College, Qingdao University, Qingdao, China; ^4^Department of Radiotherapy, Affiliated Qingdao Centre Hospital, Qingdao University, Qingdao, China; ^5^Hebei Medical University, Shijiazhuang, China; ^6^Department of Radiotherapy & Oncology, The Affiliated Suzhou Hospital of Nanjing Medical University, Gusu School, Nanjing Medical University, Suzhou, China

## Abstract

**Background:**

As a parameter integrating platelet (P), neutrophil (N), and lymphocyte (L) levels, the systemic immune-inflammation index (SII) has been used as a prognostic marker for patient survival in various types of solid malignant tumors. However, there is no in-depth study in non-small-cell lung cancer (NSCLC) patients with brain metastases after stereotactic radiotherapy. Therefore, we performed a retrospective analysis to determine the clinical and prognostic value of the SII in NSCLC patients with brain metastases who underwent stereotactic radiotherapy.

**Materials and Methods:**

We enrolled 124 NSCLC patients with brain metastases treated with stereotactic radiotherapy in our hospital between May 2015 and June 2018. We obtained all baseline blood samples within one week prior to stereotactic radiotherapy. The SII was calculated by the following formula: neutrophil counts × platelet counts/lymphocyte counts. The optimal cutoff value of the SII for predicting prognosis was assessed by receiver operating characteristic (ROC) curves with the maximum log-rank values. The discriminative ability of predicting prognosis was calculated and compared using the Kaplan–Meier method and log-rank test. The hazard ratio (HR) and 95% confidence interval (CI) were combined to evaluate the prognostic impact of the blood index on overall survival (OS) and progression-free survival (PFS). Only those parameters that proved to be associated with statistically significant differences in clinical outcomes were compared in multivariate analysis using a multiple Cox proportional hazard regression model to identify independent prognostic factors.

**Results:**

Of the total enrolled patients, 53.2% and 46.8% have high SII and low SII, respectively. In this study, Kaplan–Meier curve analysis revealed that the median PFS was 9 months (range: 2–22 months) and the median OS was 18 months (range: 4–37 months). Applying an optimal cutoff of 480 (SII), the median PFS was better in the low SII group patients (11.5 vs. 9 months), and the median OS was significantly longer in the low SII group patients (20 vs. 18 months). A SII > 480 was significantly associated with worse OS (HR: 2.196; 95% CI 1.259–3.832; *P* = 0.006) and PFS (HR: 2.471; 95% CI 1.488–4.104; *P* < 0.001) according to univariate analysis. In multivariate analysis, only age (HR: 2.159; 95% CI 1.205–3.869; *P* = 0.010), KPS (HR: 1.887; 95% CI 1.114–3.198; *P* = 0.018), and SII (HR: 1.938; 95% CI 1.046–3.589; *P* = 0.035) were independently correlated with OS, and SII (HR: 2.224; 95% CI 1.298–3.810; *P* = 0.004) was an independent prognostic predictor of PFS, whereas we found that other inflammation-based indices lost their independent value.

**Conclusions:**

The SII, which is an integrated blood parameter based on platelet, neutrophil, and lymphocyte counts, may be an independent prognostic indicator and may be useful for the identification of NSCLC patients with brain metastases after stereotactic radiotherapy at high risk for recurrence.

## 1. Introduction

Lung cancer is the most common cause of death from malignant tumors worldwide and the cancer that most frequently metastasizes to the brain during the disease course [1]. Approximately 30–50% of patients with NSCLC develop brain metastases (BMs) [2, 3]. BM represents a serious burden of illness in China and worldwide for cancer patients as a consequence of substantial effects on morbidity and quality of life. Historically, the clinical outcomes in patients with BM from NSCLC have been extremely poor. BM is usually a process of progressive deterioration, with a median survival time of 1–2 months for patients with BM without treatment [4, 5]. Optimal treatment of NSCLC with BM is controversial. Treatment of BM for NSCLC patients consists of surgical resection, radiotherapy, and epidermal growth factor receptor-tyrosine kinase inhibitors (EGFR-TKIs), which have been reported to be effective treatments [6–8]. Despite the use of advanced treatment, the clinical outcomes of NSCLC patients with BM remain poor. There is an urgent need for biomarker development and validation that allow better patient risk stratification, optimized treatment options, and prognostic prediction.

Graded Prognostic Assessment (GPA), Magnetic Resonance Spectroscopy (MRS), and Basic Score for BM (BSBM) have been considered prognostic markers of NSCLC patients with BM according to previous reports [9–11]. However, these prognostic markers provide an incomplete biological profile and cannot accurately predict the outcomes due to individual heterogeneity. Accumulating evidence has supported that inflammatory cells play an important role in the tumor microenvironment and are involved in the development of cancer, patient survival, and treatment response [12–15]. As vital parameters of the host immune-inflammatory status, platelet (P) levels, neutrophil (N) levels, lymphocyte (L) levels, neutrophil-to-lymphocyte ratio (NLR), and platelet-to-lymphocyte ratio (PLR) have been reported to be correlated with poor prognosis across many malignant tumors, including NSCLC [16–20]. As a comprehensive cancer-related inflammation hematological parameter, systemic immune-inflammation index (SII) (SII = P × N/L) could better reflect the host inflammatory and immune status balance compared to the use of a single factor or a combination of two [21, 22]. Although the SII has been shown to have independent prognostic value in multiple malignant cancers, including glioma, nasopharyngeal cancer, breast cancer, hepatocellular carcinoma, esophageal squamous cell carcinoma, gastric cancer, and prostate cancer [23–28], it has yet to be sufficiently investigated in studies involving NSCLC [22, 29, 30].

The prognostic value of the SII in NSCLC patients with BM after stereotactic radiotherapy remains unknown and needs further assessment. Hence, we conducted this retrospective study to evaluate the prognostic value of the SII in NSCLC patients who underwent stereotactic radiotherapy.

## 2. Materials and Methods

### 2.1. Study Population

**Figure 1:**
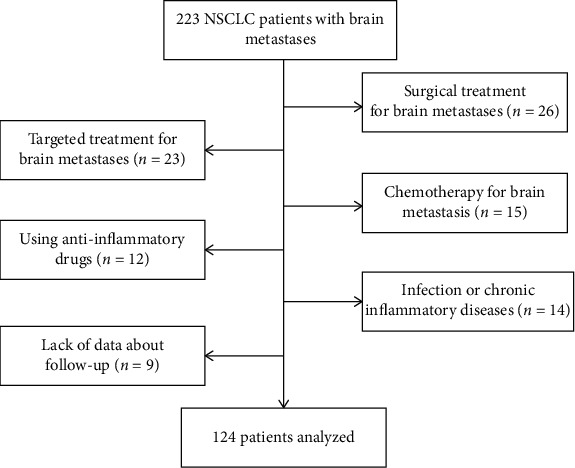
Selection of patients for this study.

This retrospective analysis enrolled 124 patients diagnosed with advanced NSCLC and BM (from May 2015 to June 2018). The hospital electronic database was used to collect the clinical data of enrolled patients. The patient flow diagram of this study is shown in Figure 1. NSCLC patients were diagnosed with BM by enhanced computed tomography (CT) or enhanced magnetic resonance imaging (MRI) during the follow-up period after individual treatment. Extracranial disease had been stably controlled. The patient eligibility criteria consisted of Karnofsky performance status (KPS) scores ranging from 70 to 100, BM number ≤ 3, histologically confirmed NSCLC, and complete demographics, hematological, and follow-up data. The patient exclusion criteria consisted of patients with serious infection or bleeding disease, chronic inflammatory disease, or autoimmune disease and those using immunosuppressive or anti-inflammatory drugs before treatment. Ninety-nine patients were excluded based on the above criteria.

### 2.2. Stereotactic Radiotherapy Regimens

**Figure 2:**
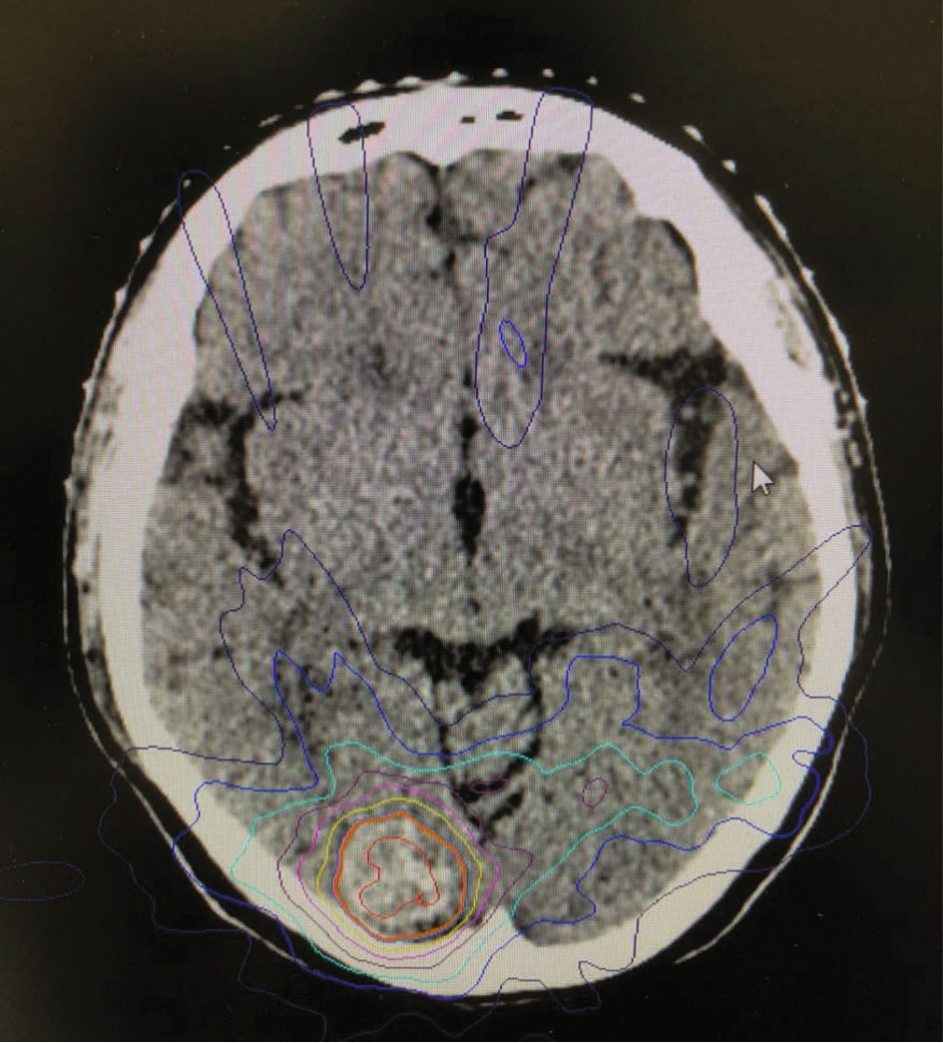
SBRT plans for brain metastasis patient. The patient was a 59-year-old female with adenocarcinoma stage II NSCLC with brain metastases in the right occipital lobe.

The injected simulation CT scanner was performed in the supine position. Planning CT scans of 1 mm thickness were acquired and fused with enhanced MRI (1 mm slice thickness) sequences of interest on a Multiplan workstation. The gross tumor volume (GTV) and the critical organ structures (brainstem, eyes, lens, optic nerve, hippocampus, and optic chiasm) were defined by CT and MRI images. When the BM location was closer to critical organ structures, the margin could be reduced to 0–1 mm, and critical organ structures were excluded from the PTV. Doses were prescribed to the 70% isodose line to achieve 99% target coverage of the PTV (Figure 2). All patients treated with cyber knife received 48 to 60 Gy in 6 to 8 fractions to the PTV based on individual physician preference. BM was irradiated on an alternate day schedule.

### 2.3. Inflammation Parameter Analysis and Follow-Up

Complete blood count data for this analysis were collected in the general laboratory of our hospital within seven days before stereotactic radiotherapy. Data on peripheral P, N, and L counts were extracted from the medical records. In the present study, we calculated the SII, NLR, and PLR as follows: SII = P × /N L, NLR = N/L, and PLR = P/L. Laboratory tests, CT, MRI, and other suitable examinations were used to confirm the recurrence, progression, or metastasis of patients by two oncologists at follow-up. Progression-free survival (PFS) was calculated from the date of stereotactic radiotherapy to the date of local recurrence or distant metastases, death, or final follow-up. Overall survival (OS) was calculated from the date of stereotactic radiotherapy to the date of death or final follow-up. Clinical follow-up was performed up to January 2019.

### 2.4. Statistical Analysis

Data analyses were conducted with GraphPad Prism (version 8.0, San Diego, CA, USA) and SPSS statistical software (version 23.0, Chicago, IL, USA). ROC curve analysis was used to determine the optimal cutoff levels for SII, NLR, and PLR when the Youden index attained the maximum value for recurrence prediction. Categorical variables were compared by chi-square or Fisher's exact test. OS and PFS differences were compared by Kaplan–Meier curves and the log-rank test. Univariate and multivariate Cox proportional hazard analyses were used to identify potential independent prognostic factors for OS and PFS. All of the reported significance levels were two-sided, and a *P* value of 0.05 or lower (*P* value < 0.05) was considered to represent statistical significance.

## 3. Results

### 3.1. Patient Characteristics

The clinical characteristics of all 124 enrolled patients are shown in Table 1. The median age was 60 years (range, 38–73 years) at the time of receiving stereotactic radiotherapy. Fifty-six (45.2%) patients were male, and 68 (54.8%) were female. Seventy patients (56.5%) had a smoking history. The majority of patients (55.6%) presented with KPS scores of 70–80. The most common histological type was adenocarcinoma (*n* = 90 (72.6%)), followed by squamous cell carcinoma (*n* = 34 (27.4%)). Most of the patients (59.7%) were diagnosed with 1 brain lesion. The median OS for the whole study population was 25 months (95% CI: 20.49–29.51 months), while the median PFS was 12 months (95% CI: 10.82–13.18 months). Median OS was 29 (95% CI: 24.76–33.24) months in patients in the low SII group, compared with a median OS of 20 (95% CI: 15.73–24.27) months in patients in the high SII group. Patients with low SII had better median PFS (17 months, 95% CI: 12.96–21.05) compared with high SII group (9 months, 95% CI: 8.04–9.96) patients. The median follow-up of all patients was 20 months (range: 6–38 months). There were 66 patients of deaths and 75 patients with any progression at the time of the final follow-up.

### 3.2. Blood Parameter and SII Analysis

**Figure 3:**
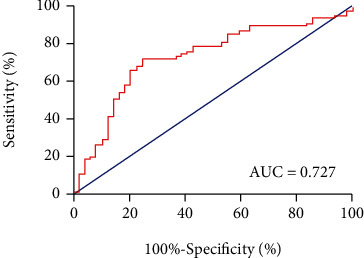
ROC curve of SII for recurrence prediction.

The clinicopathological data of patients in different SII groups are summarized in Table 2. The area under the curve (AUC) values for the neutrophil, lymphocyte, platelet, SII, NLR, and PLR were 0.540 (95% CI: 0.439-0.642), 0.436 (95% CI: 0.333-0.538), 0.592 (95% CI: 0.489-0.695), 0.727 (95% CI: 0.635-0.820), 0.593 (95% CI: 0.492-0.695), and 0.637 (95% CI: 0.535-0.738), respectively (Figure 3). ROC analysis was used to determine the optimal cutoff value for patient grouping by SII (480 × 10^9^/L), NLR (2.5 × 10^9^/L), and PLR (91.5 × 10^9^/L). With the defined optimal cutoff value, 66 (53.2%) patients were stratified into the high SII group (SII > 480) and 58 were stratified into the low SII group (SII ≤ 480). Patients in the high SII group were associated with greater smoking history (*P* < 0.001) and primary AJCC stage (*P* = 0.002), which were all considered positive prognostic factors. However, there was no significant correlation between elevated SII level and sex, age, histology type, neurologic symptoms, T stage, N stage, or number of BM.

### 3.3. Prognostic Analyses

**Figure 4:**
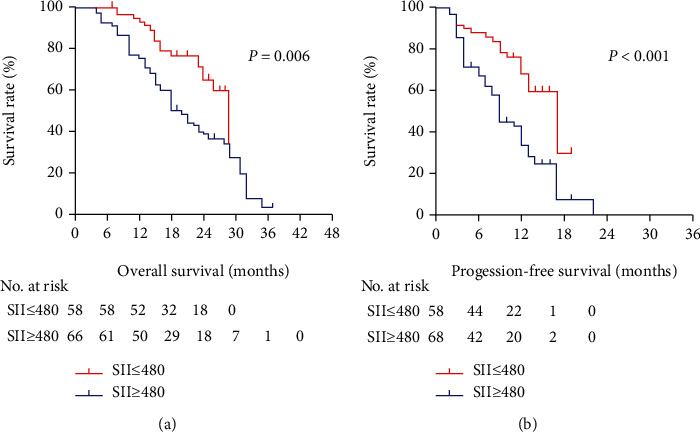
(a) Overall survival and (b) progression-free survival graphic based on SII status.

Survival curves revealed that compared with the low SII group, the high SII group had inferior survival outcomes (OS, *P* = 0.006, Figure 4(a); PFS, *P* < 0.001, Figure 4(b)). Eighteen variables were included in the univariate Cox regression analysis, and the association between the variables and survival outcome is shown in Table 3. Age (*P* = 0.002), smoking history (*P* = 0.014), KPS (*P* = 0.021), primary AJCC stage (*P* = 0.045), and SII (*P* = 0.006) were significant risk factors for OS. Histology type (*P* = 0.014), SII (*P* < 0.001), and PLR (*P* = 0.002) were significant risk factors for PFS. In multivariate Cox regression analysis, SII, NLR, and PLR were further investigated. As shown in Table 4, SII was an independent factor in predicting OS (*P* = 0.035) and PFS (*P* = 0.004), while age, smoking history, KPS, histology type, primary AJCC stage, and PLR were not considered to be independent factors.

## 4. Discussion

Despite the clinical interest in investigating the value of the SII, the clinical significance of the SII in NSCLC patients with BM remains unknown; therefore, investigating the clinical significance of the SII in NSCLC patients with BM can further deepen our understanding of immune inflammation. This study found that the SII reflects the host inflammatory status, which has prognostic value in NSCLC patients with BM. OS and PFS were significantly prolonged in the low SII group compared with the high SII group. To our knowledge, our study represents the first study to demonstrate the clinical significance of the SII in NSCLC patients with BM who underwent stereotactic radiotherapy. In this study, we demonstrated that the SII was an independent significant prognostic biomarker (OS, *P* = 0.035; PFS, *P* = 0.004).

Stereotactic radiotherapy is very often used to treat limited numbers of BMs, since this therapy is less invasive than surgical resection or supportive care [31, 32]. Nevertheless, the clinical outcomes for NSCLC patients after stereotactic radiotherapy remain poor. Early assessment of reliable prognosis can guide clinical decision-making and improve patient survival and quality of life. Therefore, it is crucial to have a comprehensive understanding of an individual patient's risk of experiencing progression or death. Some studies have substantiated the association between malignant tumors and the inflammatory system [33]. Immune and inflammatory cells (neutrophils, platelets, and lymphocytes) can regulate the balance of the host inflammatory and immune status, which are associated with prognostic value in multiple tumor types [34–38]. Several mechanisms can potentially explain why peripheral blood parameters statistically affect OS or PFS in cancer patients. First, neutrophils are inflammatory and immune parameters that play a role in tumor development, progression, and distant metastasis by restraining inflammatory mediators, such as matrix metalloproteinase-9, interleukin-8, neutrophil elastase, and vascular endothelial growth factor [39–41]. Second, platelets can release proangiogenic proteins and platelet-derived growth factors to promote the migration and angiogenesis of tumor cells [42]. Moreover, platelets can directly act as protective “cloaks” to shield CTCs from immune attack, induce epithelial-mesenchymal transition, and facilitate extravasation and metastasis of tumor cells [43]. As a result, elevated platelet levels are positively associated with poor survival in cancer. Third, lymphocytes, as immune guards, play an important role in systematic immune surveillance and immune injury by inducing cytotoxic cell death and cytokine secretion [44, 45]. In addition, a high lymphocyte level is associated with better clinical outcomes in solid tumors, possibly because the host's antitumor immune response is strengthened as lymphocyte levels increase [46, 47].

The SII, including neutrophils, platelets, and lymphocytes, is based on three types of immune and inflammatory cells. The SII should be a more objective valid surrogate that reflects the balance between host immune and inflammatory status compared to other systemic immune-inflammation scores. To date, the role of the SII in prognosis has been investigated in patients with hepatocellular carcinoma [25], pancreatic cancer [21], small cell lung cancer [48], gastric cancer [23], classical Hodgkin lymphoma [49], esophageal squamous cell carcinoma [50], and breast cancer [51]. Few studies have investigated the prognostic role of the SII in NSCLC. Yucel and Bilgin evaluated the prognostic role of the SII in EGFR-mutant advanced NSCLC. The results demonstrated that a low SII was significantly correlated with better OS (32.4 vs. 20.4 months; *P* = 0.005) and PFS (22.4 vs. 13.01 months, *P* = 0.003) [52]. Yan et al. conducted a meta-analysis that investigated the prognostic role of the SII in NSCLC. A total of 2441 patients were eventually included in their study, and an elevated SII indicated significantly poorer OS (HR = 1.88, *P* < 0.001) [36]. Furthermore, other findings in the field of immunotherapy [53] suggested that the SII is independently associated with PFS and OS in patients with metastatic NSCLC treated with nivolumab. However, the SII has not been evaluated in the setting of stereotactic radiotherapy for NSCLC patients with BM to date, and our study may help to address this issue. In this study, we compared several immune-inflammation parameters (SII, NLR, and PLR), and our results demonstrate the superiority of the SII for predicting the prognosis in NSCLC patients with BM. In concomitance with other studies, the SII plays a role in major metastatic cancer types, including NSCLC. In particular, patients with high SII were related to shorter OS (median OS 18 vs. 20 months) and PFS (median PFS 9 vs. 11.5 months). The SII maintained its value in univariate and multivariate analyses for clinical outcomes, indicating the status of the host inflammatory and immune status. SII was independently associated with OS (*P* = 0.035, HR: 1.938, 95% CI: 1.046–3.589) and PFS (*P* = 0.004, HR: 2.224, 95% CI: 1.298–3.810), and the risk of progression or death was higher, approximately 50%, in the high SII group than in the low SII group.

We acknowledge that our study had some limitations, despite its promising results, which may limit interpretation of the findings. First, this was a retrospective single-center analysis of a relatively smaller study population that is subject to selection biases. Second, it is difficult to compare our cutoff points of SII with those of other studies because of heterogeneous differences in individual studies. Third, although this is the first study that evaluated the prognostic value of the SII in NSCLC patients with BM after stereotactic radiotherapy, we lacked external study validation. Further prospective and large-scale studies are needed to validate the results of our study.

## 5. Conclusion

Taken together, we can draw conclusions regarding the value of the SII on survival based on the results of our study. This is the first study to demonstrate that the SII could represent an independent prognostic factor in NSCLC patients with BM treated with stereotactic radiotherapy.

## Figures and Tables

**Table 1:** Patient characteristics.

Parameters	*N* (%)
Sex	
Male	56 (45.2)
Female	68 (54.8)
Age (years)	
<60	59 (47.6)
≥60	65 (52.4)
Smoking history	
Never smoker	54 (41.5)
Smoker	70 (56.5)
KPS	
90-100	55 (44.4)
70-80	69 (55.6)
Histology type	
SCC	34 (27.4)
AD	90 (72.6)
Differentiation	
Well	21 (16.9)
Moderate	32 (25.8)
Poor	71 (57.3)
Primary site of tumor	
Right	66 (53.2)
Left	58 (46.8)
Neurologic symptoms	
No	60 (48.4)
Yes	64 (51.6)
Primary T stage	
T1	36 (29.0)
T2	41 (33.1)
T3	18 (14.5)
T4	29 (23.4)
Primary N stage	
N0	42 (33.9)
N1	45 (36.3)
N2	22 (17.7)
N3	15 (12.1)
Primary AJCC stage	
I	87 (70.2)
II	16 (12.9)
III	21 (16.9)
No. of BM	
1	74 (59.7)
2	27 (21.8)
3	23 (18.5)
SII	
≤480	58 (46.8)
>480	66 (53.2)
NLR	
≤2.5	51 (41.1)
>2.5	73 (58.9)
PLR	
≤91.5	44 (35.5)
>91.5	80 (64.5)

Abbreviations: KPS: Karnofsky performance status; SCC: squamous cell carcinoma; AD: adenocarcinoma; BM: brain metastasis; SII: systemic immune-inflammation index; NLR: neutrophil-to-lymphocyte ratio; PLR: platelet-to-lymphocyte ratio.

**Table 2:** The clinicopathological characteristics of NSCLC patients according to SII status.

Parameters	SII (*n* = 124), *n* (%)		*P* value
	≤480 (*n* = 58)	>480 (*n* = 66)	
Sex			0.944
Male	26 (44.8)	30 (45.5)	
Female	32 (55.2)	36 (54.5)	
Age (years)			0.613
<60	29 (50.0)	30 (45.5)	
≥60	29 (50.0)	36 (54.5)	
Smoking history			<0.001
Never smoker	35 (60.3)	19 (28.8)	
Smoker	23 (39.7)	47 (71.2)	
KPS			0.644
90-100	27 (46.6)	28 (42.4)	
70-80	31 (53.4)	38 (57.6)	
Histology type			0.398
SCC	18 (41.4)	16 (24.2)	
AD	40 (58.6)	50 (75.8)	
Differentiation			0.660
Well/moderate	26 (44.8)	27 (40.9)	
Poor	32 (55.2)	39 (59.1)	
Primary site of tumor			0.963
Right	31 (53.4)	35 (53.0)	
Left	27 (46.6)	31 (47.0)	
Neurologic symptoms			0.290
No	31 (53.4)	29 (43.9)	
Yes	27 (46.6)	37 (56.1)	
Primary T stage			0.454
T1-T2	34 (58.6)	43 (65.2)	
T3-T4	24 (41.4)	23 (34.8)	
Primary N stage			0.065
N0-N1	36 (62.1)	51 (77.3)	
N2-N3	22 (37.9)	15 (22.7)	
Primary AJCC stage			0.002
I	40 (69.0)	27 (40.9)	
II-III	18 (31.0)	39 (59.1)	
No. of BM			0.091
1	30 (51.7)	44 (66.7)	
2-3	28 (48.3)	22 (33.3)	

Abbreviations: KPS: Karnofsky performance status; SCC: squamous cell carcinoma; AD: adenocarcinoma; BM: brain metastasis; SII: systemic immune-inflammation index.

**Table 3:** Univariate Cox regression analyses of the SII for OS and PFS in NSCLC patients with BM.

Parameters	OS		PFS	
	HR (95% CI)	*P* value	HR (95% CI)	*P* value
Sex				
Male	Reference	0.935	Reference	0.973
Female	0.980 (0.600-1.601)		0.992 (0.628-1.567)	
Age (years)				
<60	Reference	0.002	Reference	0.075
≥60	2.273 (1.343-3.845)		1.531 (0.959-2.447)	
Smoking history				
Never smoker	Reference	0.014	Reference	0.062
Smoker	1.982 (1.148-3.421)		1.579 (0.978-2.548)	
KPS				
90-100	Reference	0.021	Reference	0.275
70-80	1.837 (1.094-3.085)		1.295 (0.814-2.062)	
Histology type				
SCC	Reference	0.818	Reference	0.014
AD	1.067 (0.613-1.857)		2.100 (1.165-3.784)	
Differentiation				
Well/moderate	Reference	0.072	Reference	0.242
Poor	1.640 (0.956-2.814)		1.337 (0.822-2.175)	
Primary site of tumor				
Right	Reference	0.815	Reference	0.422
Left	0.944 (0.579-1.537)		1.206 (0.763-1.904)	
Neurologic symptoms				
No	Reference	0.209	Reference	0.652
Yes	0.730 (0.447-1.193)		0.900 (0.569-1.423)	
Primary T stage				
T1-T2	Reference	0.057	Reference	0.737
T3-T4	0.584 (0.336-1.016)		0.921 (0.571-1.488)	
Primary N stage				
N0-N1	Reference	0.344	Reference	0.946
N2-N3	0.761 (0.432-1.340)		0.983 (0.592-1.632)	
Primary AJCC stage				
I	Reference	0.045	Reference	0.299
II-III	1.689 (1.012-2.820)		1.276 (0.806-2.020)	
No. of BM				
1	Reference	0.680	Reference	0.798
2-3	0.899 (0.544-1.488)		0.941 (0.589-1.503)	
Neutrophil				
≤6.1	Reference	0.056	Reference	0.156
>6.1	1.894 (1.125-3.189)		1.452 (0.867-2.431)	
Lymphocyte				
≤3.5	Reference	0.521	Reference	0.658
>3.5	1.912 (0.263-13.879)		0.638 (0.087-4.671)	
Platelet				
≤169	Reference	0.506	Reference	0.129
>169	1.193 (0.709-2.009)		1.481 (0.892-2.457)	
SII				
≤480	Reference	0.006	Reference	<0.001
>480	2.196 (1.259-3.832)		2.471 (1.488-4.104)	
NLR				
≤2.5	Reference	0.336	Reference	0.343
>2.5	1.290 (0.768-2.167)		1.258 (0.783-2.021)	
PLR				
≤91.5	Reference	0.177	Reference	0.002
>91.5	1.456 (0.843-2.515)		2.360 (1.355-4.109)	

Abbreviations: BM: brain metastasis; OS: overall survival; PFS: progression-free survival; HR: hazard ratio; CI: confidence interval; KPS: Karnofsky performance status; SCC: squamous cell carcinoma; AD: adenocarcinoma; SII: systemic immune-inflammation index; NLR: neutrophil-to-lymphocyte ratio; PLR: platelet-to-lymphocyte ratio.

**Table 4:** Multivariate Cox regression analyses of the SII for OS and PFS in NSCLC patients with BM.

Parameters	OS		PFS	
	HR (95% CI)	*P* value	HR (95% CI)	*P* value
Age (years)				
<60	Reference	0.010		
≥60	2.159 (1.205-3.869)			
Smoking history				
Never smoker	Reference	0.493		
Smoker	1.233 (0.677-2.246)			
KPS				
90-100	Reference	0.018		
70-80	1.887 (1.114-3.198)			
Histology type				
SCC			Reference	0.153
AD			1.690 (0.822-3.473)	
Primary AJCC stage				
I	Reference	0.939		
II-III	1.023 (0.576-1.816)			
SII				
≤480	Reference	0.035	Reference	0.004
>480	1.938 (1.046-3.589)		2.224 (1.298-3.810)	
PLR				
≤91.5			Reference	0.392
>91.5			1.362 (0.671-2.766)	

Abbreviations: BM: brain metastasis; OS: overall survival; PFS: progression-free survival; HR: hazard ratio; CI: confidence interval; KPS: Karnofsky performance status; SCC: squamous cell carcinoma; AD: adenocarcinoma; SII: systemic immune-inflammation index; PLR: platelet-to-lymphocyte ratio.

## Data Availability

The data used to support to the findings of this study are available from the corresponding author upon request.
